# Clinical characteristics and phenotypes of inflammatory bowel disease in Northern Central America: initial results from the Northern Central America Inflammatory Bowel Disease Registry

**DOI:** 10.1093/crocol/otag106

**Published:** 2026-10-21

**Authors:** Eleazar E Montalvan-Sanchez, Michael Dougherty, Dalton Norwood, Aida Rodriguez-Murillo, Kathya Jimenez, Daniela M Montalvan-Sanchez, Luis A Najera, Blanca A Avalos-Quijano, Oscar G Avelar-Escobar, Marco A Calix-Cerrato, Gustavo A Ramos-Aguilar, Daniel Guifarro, Jeffrey Freidenson, Candida Leiva, Douglas Morgan, Deborah Proctor, Alexander N Levy

**Affiliations:** Section of Digestive Diseases, Department of Medicine, Yale School of Medicine, New Haven, CT, United States; Division of Gastroenterology and Hepatology, Department of Medicine, University of North Carolina School of Medicine, Chapel Hill, NC, United States; Division of General Internal Medicine, University of Alabama at Birmingham, Birmingham, AL, United States; Department of Medicine, University of Nevada Reno, Reno, NV, United States; Department of Medicine, University of Nevada Reno, Reno, NV, United States; Departamento de Medicina Interna, Universidad Autonoma de Honduras, Tegucigalpa, Honduras; Departamento de Medicina Interna, Hospital General San Juan de Dios, Ciudad de Guatemala, Guatemala; Department of Medicine, Universidad Evangelica de el Salvador, San Salvador, El Salvador; Instituto Salvadoreño del Seguro Social El Salvador, San Salvador, El Salvador; Secretaria de Salud de Honduras, Tegucigalpa, Honduras; Honduras Medical Center, Tegucigalpa, Honduras; Department of Medicine, Cook County Health, Chicago, IL, United States; Department of Medicine, Indiana University School of Medicine, Indianapolis, IN, United States; Departamento de Medicina Interna, Hospital Roosevelt, Ciudad de Guatemala, Guatemala; Division of Gastroenterology and Hepatology, University of Alabama at Birmingham, Birmingham, AL, United States; Section of Digestive Diseases, Department of Medicine, Yale School of Medicine, New Haven, CT, United States; Section of Digestive Diseases, Department of Medicine, Yale School of Medicine, New Haven, CT, United States

**Keywords:** inflammatory bowel disease, epidemiology, Central America

## Abstract

**Background:**

Inflammatory bowel disease (IBD), encompassing ulcerative colitis (UC), Crohn’s disease (CD), and IBD unclassified (IBDU), affects over 7 million people worldwide. While incidence has plateaued in higher-income countries, it continues to rise in lower- and middle-income regions, including Latin America. Data from northern Central America remain scarce.

**Aim:**

To characterize the clinical and phenotypic profile of IBD in Honduras, El Salvador, and Guatemala.

**Methods:**

The Inflammatory Bowel Disease in Northern Central America Research and Epidemiological Study (IBD-NCARES) was a multinational, multicenter descriptive study spanning 15 years. Cases were identified using ICD-10 codes and confirmed by chart review. Sociodemographic, behavioral, and clinical variables were collected.

**Results:**

Among 277 patients, UC was the predominant subtype (57.8%), highest in Honduras (71.4%). CD accounted for 11.9% of cases, with greater frequency in Honduras (25.7%). IBDU was frequent in El Salvador (40.3%) and Guatemala (38.2%) compared with Honduras (2.9%). Delayed diagnosis (>1 year) occurred in 35% of UC and 60.6% of CD patients. Complicated CD was observed in 54.5% (B2 30.3%; B3 24.2%). Extraintestinal manifestations included vascular (18.2%) and hepatic/pancreatic (9.1%) complications. Most patients received 5-aminosalicylates or systemic steroids. Only 5.1% of the overall cohort received advanced therapies.

**Conclusion:**

This first multinational study of IBD in northern Central America highlights UC predominance, high rates of IBDU (likely inflated by incomplete diagnostics), frequent diagnostic delay, and limited use of advanced therapies. These findings underscore the need for regional registries, improved diagnostic capacity, and expanded therapeutic resources.

## Graphical Abstract

**Graphical Abstract:**
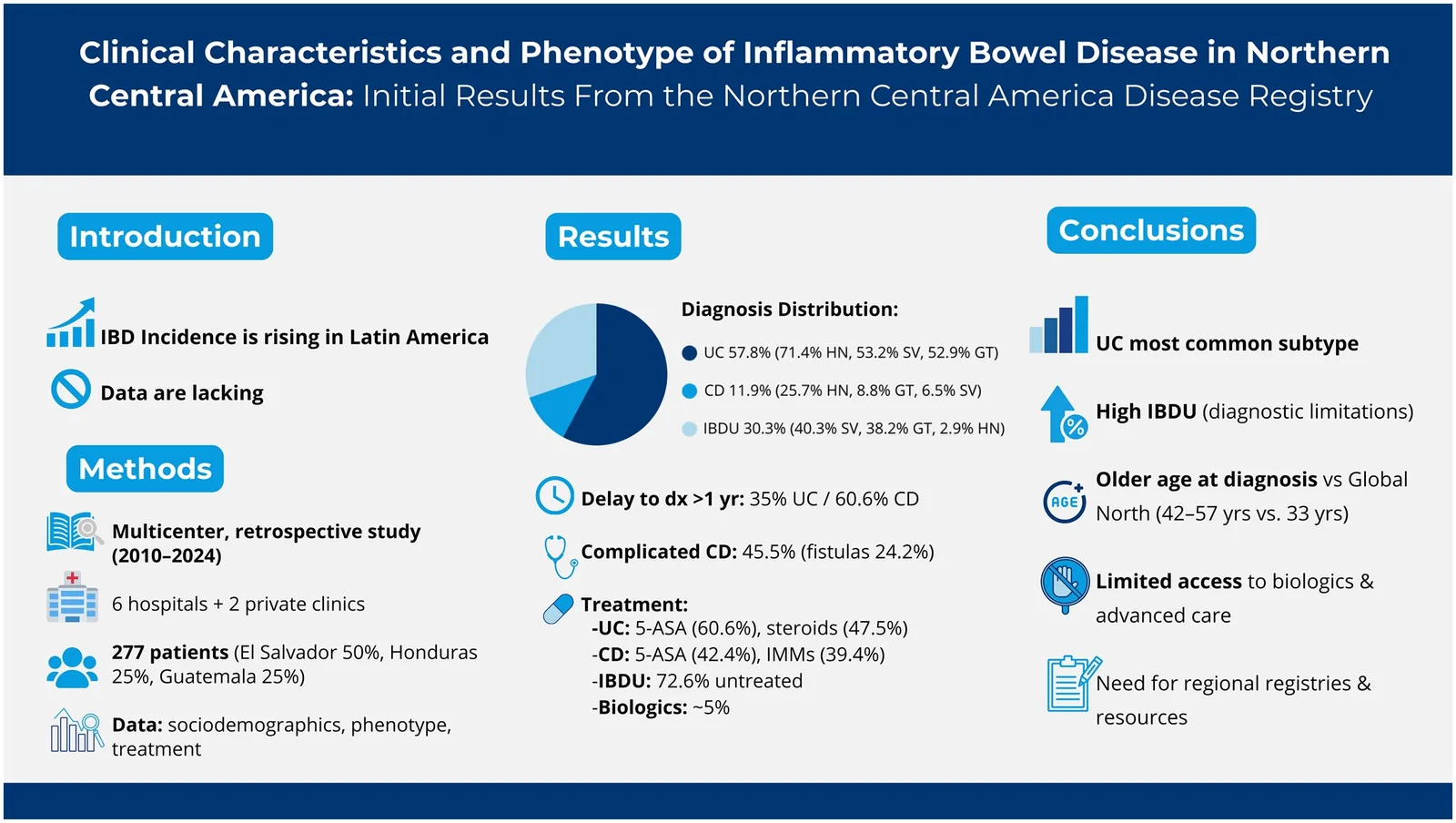

## Introduction

Inflammatory bowel disease (IBD), which includes ulcerative colitis (UC) and Crohn’s disease (CD), affects over 7 million people worldwide.^1^^,^^2^ The global prevalence of IBD varies significantly across different countries, with higher-income countries in the Global North^3^ having the highest prevalence of IBD.^4^ Although incidence rates in the Global North have stabilized over the past decade,^4^ rising incidence in lower- and middle-income countries (LMICs) in the Global South are driving prevalence higher, particularly in countries with rapid development, such as Latin America and the Caribbean.^5^ The exact pathophysiological underpinnings of IBD remain unclear;^6^^,^^7^ however, the substantial increases in IBD in rapid growth countries suggest that environmental factors, industrialization, and rapid socioeconomic development may precede increases in IBD burden.^1^^,^^8^^,^^9^^,^^10^

Despite recent data demonstrating the increased incidence of IBD in the Global South, few population-based studies have been published characterizing the features of IBD in Latin America, particularly in LMICs. The lack of epidemiological data in other LMIC countries in northern Central America, such as El Salvador, Guatemala, Honduras, and Nicaragua, constitutes a gap in current medical knowledge and a barrier to addressing health disparities in this region.^5^^,^^11^ Healthcare access across Northern Central America is highly heterogeneous and often constrained by limited diagnostic and therapeutic resources. In Honduras, Guatemala, and El Salvador, colonoscopy capacity is frequently restricted to tertiary referral centers and emergency indications. Histopathologic evaluation and advanced imaging such as CT and MRI are not consistently available and often require out-of-pocket payment by patients. Access to biologic therapies for IBD is extremely limited within public healthcare systems and is typically available only through private practice settings.

Understanding the clinical features of IBD in Central America could provide clues as to which environmental risk factors contribute to IBD development. Such knowledge would also be potentially applicable to Latinos/Hispanics living in the United States (U.S.), the largest ethnic minority population in the country.^12^ The present study aimed to characterize the clinical presentation, diagnostic patterns, disease phenotype, and treatment utilization of patients with IBD in Northern Central America using multicenter real-world clinical data.

## Methods

The Inflammatory Bowel Disease in Northern Central America Research and Epidemiological Study (IBD-NCARES) was a retrospective, multinational study conducted across 6 hospitals and gastroenterology private practices in Honduras, El Salvador, and Guatemala between 2010 and 2024. Participating sites included national tertiary care hospitals with gastrointestinal services in Guatemala City (Hospital Roosevelt and San Juan Hospital), San Salvador (El Salvador Institute of Social Security), Tegucigalpa, Honduras (Hospital Escuela Universitario), Santa Rosa de Copan, Honduras (Hospital de Occidente), and San Pedro Sula, Honduras (Hospital Mario Catarino Rivas), as well as two gastroenterology private practices in San Salvador, El Salvador, and Tegucigalpa, Honduras.

### Case identification

IBD cases were screened using ICD-10 codes (K50 for CD, K51 for UC, and K52 for IBD unclassified [IBDU]). Owing to insufficient coding in Central American countries, an additional screen was performed using a keyword search of endoscopy and histology reports (Supplementary Table 1). Initial case identification was performed locally by gastroenterologists and endoscopists at each participating center. Diagnostic verification was subsequently conducted through centralized chart review by a panel consisting of two gastroenterology fellows (Candy Leiva and Luis A. Najera) and two senior gastroenterologists (Gustavo A. Ramos-Aguila and Oscar G. Avelar-Escobar). Medical records of all screened cases were reviewed to confirm the IBD diagnosis and cross-check for duplication. Diagnostic criteria for IBD included evidence of typical inflammation on endoscopy, colonoscopy, histology, and/or imaging. Specifically, a diagnosis of IBDU (indeterminate colitis) was applied when, despite full colonoscopy, UC could not be distinguished from CD due to atypical features on endoscopic appearance, histopathology, or both, and no clinical or radiologic evidence was available to suggest CD (such as CT evidence of involvement proximal to the terminal ileum or perianal disease). The reviewers ratified the local diagnoses except when there was insufficient documentation to support an IBD diagnosis or exclude alternative diagnoses, in which case these were excluded. A flow diagram illustrating case identification, screening, and inclusion is shown in Figure 1.

**Figure 1:**
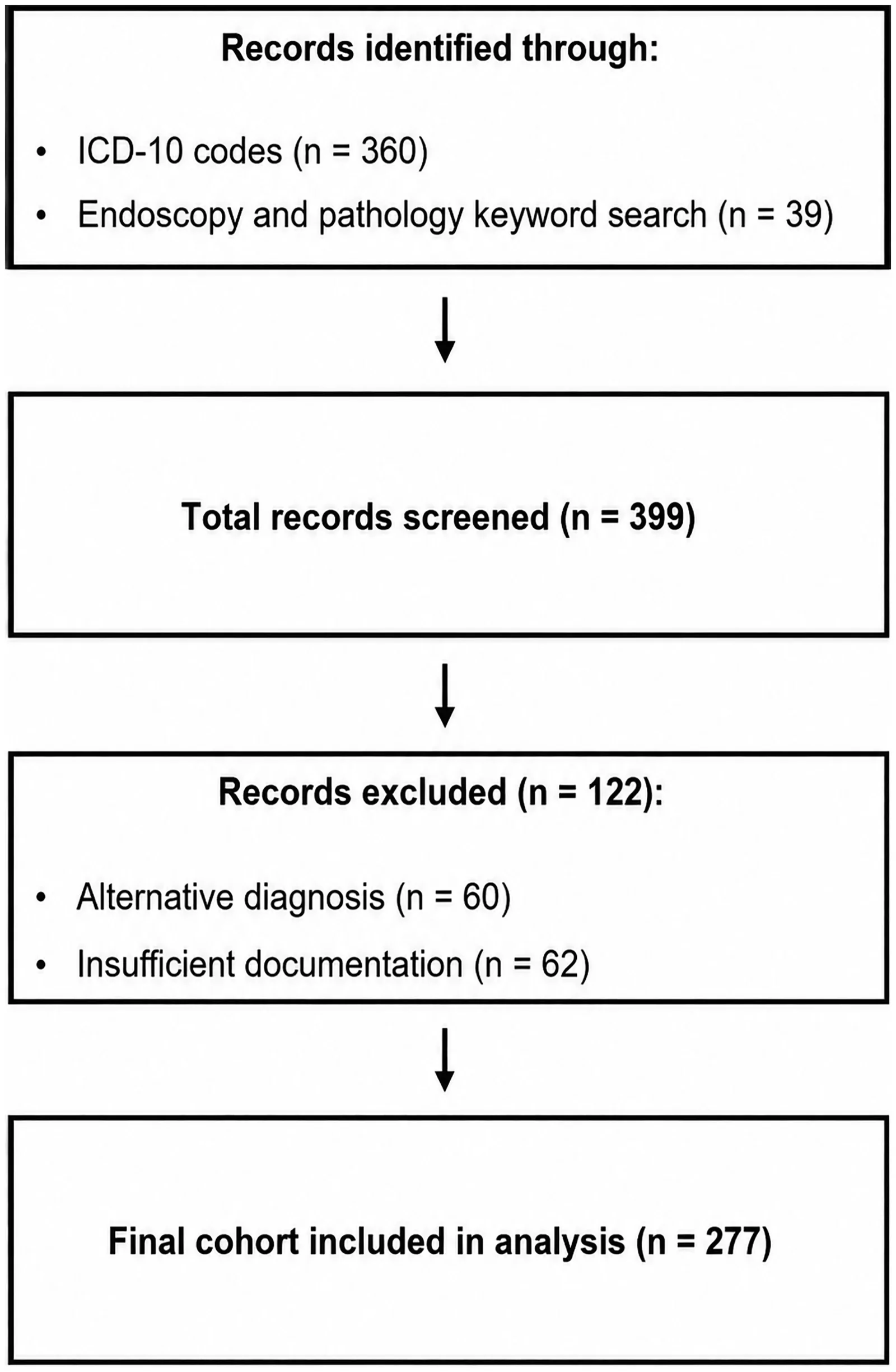
Flow diagram illustrating case identification, screening, exclusions, and final inclusion of patients with inflammatory bowel disease across participating centers. ICD-10, International Classification of Diseases, 10th Revision.

### Definition of variables

The variables collected included sociodemographic information such as age, sex, marital status, education level, and nationality. Additionally, we collected data on IBD subtype, disease duration, disease severity, time to diagnosis, and medication use. Disease severity was assessed using the Montreal and Paris classifications for CD and UC. Previously identified risk factors such as sedentarism (defined as expending less than 10% of daily energy in the performance of moderate- and high-intensity activities), alcohol consumption, smoking status, and tuberculosis exposure, were also captured. All utilized data was translated from Spanish by bilingual medical staff and verified by two certified medical translators.

Because of variability in documentation across participating centers and the retrospective nature of the study, standardized disease activity indices such as the Mayo score or SES-CD were not consistently available. The only consistently available laboratory markers were hemoglobin, leucocytes, and platelets.

All utilized data was translated from Spanish by bilingual medical staff and verified by 2 certified medical translators. Following translation, data was extracted and recorded into an electronic data capture system (REDCap),^13^ allowing for standardized data collection. The data were extracted between June 2021 and February 2024.

### Statistical analyses

Our primary aim was to characterize the phenotype of IBD in LMICs, according to categories of UC, CD, and IBDU. Descriptive statistics were reported as means, medians, and proportions. Comparative frequencies and distributions were segmented according to the type of diagnosis and country. Statistical analyses were conducted using STATA 18.^14^ The study protocol was approved by the ethics committees of the participating hospitals.

## Results

In total, 277 patients with IBD were included in the analysis. Temporal trends were evaluated by comparing cases diagnosed across 3 quinquennia (2010-2014, 2015-2019, and 2020-2024). UC remained the predominant diagnosis across all periods but declined from 67.4% in the earliest period to 56.8% in 2015-2019 and 57.3% in 2020-2024. CD increased from 9.3% to 16.7%, while IBDU peaked at 32.8% in 2015-2019 before decreasing to 26.0% in the most recent period (Table S4). The sample represented the majority of the tertiary care referral populations for each country. El Salvador contributed half of the study patients (139, 50.2%), the majority coming from private practice (97/139), while Guatemala (68, 24.5%), and Honduras (70, 25.3%) each contributed one-quarter of study patients. The distribution of patients by diagnosis type was 160 patients with UC (57.8%), 33 patients with CD (11.9%), and 84 patients with IBDU (30.3%), though only 35 IBDU patients had histopathology available to sufficiently exclude definitive UC or CD.

The distribution of diagnoses varied significantly by country and practice setting. UC was the most common diagnosis in each country, comprising 53.2% of diagnoses in El Salvador, 52.9% in Guatemala, and 71.4% in Honduras. Honduras had the highest proportion of patients classified as CD (25.7%), while patients in El Salvador and Guatemala were more likely to be classified as IBDU (40.3% and 38.2%, respectively) and uncommonly applied a diagnosis of CD (6.5% and 8.8%, respectively, Supplementary Table 2). With regard to practice setting, in El Salvador UC was the most frequent diagnosis in hospital-based patients (37/42 patients, Table 1). UC was still common in Salvadoran private clinics (37/97 private patients), but IBDU was the most common diagnosis (56/97 private clinic patients) and, in fact, was only diagnosed in this setting (no Salvadoran hospital-based patients were diagnosed with IBDU). CD was uncommon in both Salvadoran clinical settings (5/42 and 4/97 patients). In Honduras, there were only 12 patients diagnosed in private clinics, all with UC, though UC was still the most frequent diagnosis in hospital-based settings (38/58 patients, 65.5%), with 18 patients diagnosed with CD, and only 2 IBDU. In Guatemala, there were no private clinic patients to compare, so all IBDU cases were diagnosed in the hospital-based setting. The higher proportion of IBDU cases in private clinics in El Salvador was significantly different from that of the other countries (*P*<.001).

**Table 1:** Table of general characteristics of the population by diagnosis type.

Diagnosis type	UC	CD	IBDU	*P*-value
	160 (57.8%)	33 (11.9%)	84 (30.3%)	
** El Salvador**	74 (46.2%)	9 (27.3%)	56 (66.7%)	<.001
** Academic centers**	37 (50%)	5 (56%)	0 (0%)	
** Private practice**	37 (50%)	4 (44%)	56 (100%)	
** Guatemala**	36 (22.5%)	6 (18.2%)	26 (30.9%)	NA
** Academic centers**	36 (100%)	6 (100%)	26 (100%)	
** Private practice**	0 (0%)	0 (0%)	0 (0%)	
**Honduras**	50 (31.2%)	18 (54.5%)	2 (2.4%)	.055
** Academic centers**	38 (76%)	18 (100%)	2 (100%)	
** Private practice**	12 (24%)	0 (0%)	0 (0%)	
**Age at diagnosis, median (IQR)**	42.3 (30.0, 54.2)	47.8 (38.7, 62.9)	56.9 (40.0, 64.6)	<.001
** <18**	13 (9.0%)	3 (9.4%)	2 (2.4%)	
** 18-24**	15 (10.3%)	1 (3.1%)	4 (4.9%)	
** 25-44**	52 (35.9%)	8 (25.0%)	19 (23.2%)	
** 45-64**	46 (31.7%)	14 (43.8%)	38 (46.3%)	
** > =65**	19 (13.1%)	6 (18.8%)	19 (23.2%)	
**Sex**				.043
** Female**	89 (56.3%)	12 (37.5%)	52 (63.4%)	
** Male**	69 (43.7%)	20 (62.5%)	30 (36.6%)	
**Marital Status**				<.001
** Married or living together**	65 (40.6%)	15 (45.5%)	18 (21.4%)	
** Single**	56 (35.0%)	8 (24.2%)	9 (10.7%)	
** Widowed**	4 (2.5%)	1 (3.0%)	0 (0.0%)	
** Other/Unknown**	35 (21.9%)	9 (27.3%)	57 (67.9%)	
**Patient residence Urban**	115 (71.9%)	28 (84.8%)	40 (47.6%)	<.001
** Rural**	45 (28.1%)	5 (15.2%)	44 (52.4%)	
**Sedentarism**	96 (61.9%)	17 (51.5%)	55 (66.3%)	.34
**Alcohol consumption**	24 (15.0%)	9 (27.3%)	15 (17.9%)	.23
**Tobacco (ever)**	37 (23.1%)	3 (9.1%)	8 (9.5%)	.012
**Comorbidities**				
** Cardiovascular**	24 (15.0%)	9 (27.3%)	28 (33.3%)	.003
** Metabolic**	23 (14.4%)	5 (15.2%)	11 (13.1%)	.95
** Infectious**	4 (2.5%)	1 (3.0%)	3 (3.6%)	.89
** Chronic/non-infectious**	28 (17.5%)	10 (30.3%)	2 (2.4%)	<.001
** Neurological**	1 (0.6%)	1 (3.0%)	0 (0.0%)	.21
** Cancer**	3 (1.9%)	0 (0.0%)	3 (3.6%)	.45
** Other autoimmune**	6 (3.8%)	0 (0.0%)	0 (0.0%)	.11
** None**	83 (51.9%)	5 (15.2%)	43 (51.2%)	<.001
**History of tuberculosis**	3 (1.9%)	0 (0.0%)	3 (3.6%)	.454

Baseline demographic and clinical features of patients with ulcerative colitis (UC), Crohn’s disease (CD), and IBD-unclassified (IBDU) across El Salvador, Guatemala, and Honduras. Data include age at diagnosis, sex, marital status, geographic setting, lifestyle factors, comorbidities, and history of tuberculosis. Stratification by academic centers vs. private practice is shown. Eighteen (*n* = 18) had missing age-at-diagnosis data; percentages were calculated using available data (UC *n* = 15, CD *n* = 1, IBDU *n* = 1). Comorbidities are not mutually exclusive; patients may contribute to more than one comorbidity and report missing/unknown status, each *P*-value compares presence versus absence for that row.

Regarding the diagnosis of IBDU, essentially indeterminate colitis, most patients were not classified as UC due to atypical/discordant endoscopic and/or histologic features. Due to limited availability at many of the sites, full workups, including histopathology examination and cross-sectional imaging were not always available to help further distinguish between subtypes of IBD, and likely contributed to the higher percentages of IBDU than in higher-resource settings. To focus on those patients with a more truly “indeterminate” colitis, we performed a sensitivity analysis restricting the IBDU diagnosis to those with histopathologic results (Table S6). No significant numerical or statistical differences were observed.

### Sociodemographic and behavioral risk factors

Sociodemographic and medical history data are presented in Table 1. The median age at diagnosis differed significantly among the three diagnoses (*P*<.001). Patients with UC was a median age at diagnosis of 42.3 years (IQR: 30.0-54.2), patients with CD had a median age of 47.8 years (IQR: 38.7-62.9), and patients with IBDU had a median age of 56.9 years (IQR: 40.0-64.6). The sex distribution also differed significantly among the 3 diagnostic groups (*P*=.043), with the highest percentage of females in the IBDU group (63.4%), followed by UC (56.3%), and finally CD (37.5%).

Significant geographic differences were observed among diagnosis groups (*P*<.001), with the majority of UC and CD patients receiving care in urban areas (71.9%, 84.8%, respectively), while IBDU patients were more evenly divided (47.6% urban). These did not differ by practice type, as all of the private clinics as well as the hospitals were urban. There were significant differences in reported education levels between groups, however, a substantial percentage had unknown educational status. No significant differences were found in sedentarism or alcohol consumption among the diagnosis groups. Tobacco use differed significantly (*P*=.012), with the lowest proportion of smokers observed in the CD group (9.1%) compared to UC (23.1%). Men (29.8%, 23.1%, 34.9%) had a higher prevalence of current smoking than women (3.1%, 2.6%, 4.4%) in Guatemala, Honduras, and El Salvador, respectively.

### Disease behavior

The distribution of disease in CD, based on the Montreal classification, comprised 15.2% isolated terminal ileum involvement (L1), 27.3% colon involvement (L2), 33.3% ileo-colonic involvement (L3), and 3.0% upper digestive tract involvement (L4). Regarding disease behavior, 45.5% had non-stricturing and non-penetrating disease (B1), 30.3% had stricturing disease (B2), and 24.2% had penetrating disease (B3). Only 6.1% of CD patients had perianal involvement. In UC, the distribution was as follows (Paris classification): 25.6% had proctitis (E1), 25% had left-sided involvement (E2), 10% had extensive involvement (E3), and 12.5% had pancolitis (E4). Disease severity classification revealed 57.5% had never-severe disease and 11.2% had ever-severe disease (Table 2).

**Table 2:** Distribution of patients according to the Montreal classification for Crohn’s and the Paris classification for UC.

**Crohn’s Disease**	
** N**	*N* = 33
** Montreal classification: Location**	
** L1: Terminal ileum**	5 (15.2%)
** L2: Colon**	9 (27.3%)
** L3: Ileo-colon**	11 (33.3%)
** L4: Upper digestive track**	1 (3.0%)
** Missing/Unknown**	7 (21%)
** Montreal classification B1: Non-stricturing and non-penetrating**	15 (45.5%)
** Montreal classification B2: Stricturing**	10 (30.3%)
** Montreal classification B3: Penetrating**	8 (24.2%)
** Montreal classification p: Perianal**	2 (6.1%)
**Ulcerative colitis**	
** N**	*N* = 160
** Paris classification**	
** E1: Proctitis**	41 (25.6%)
** E2: Left-sided (and distal to the splenic flexure)**	40 (25.0%)
** E3: Extensive (and distal to the hepatic flexure)**	16 (10.0%)
** E4: Pancolitis (proximal to the hepatic flexure)**	20 (12.5%)
** Missing/Unknown**	43 (26.9%)
** Paris severity classification**	
** Never severe**	92 (57.5%)
** Ever severe**	18 (11.2%)
** Missing/Unknown**	50 (31.3%)

Phenotypic distribution of Crohn’s disease (*n* = 33) based on Montreal location and behavior classifications (L1-L4, B1-B3, perianal disease) and of ulcerative colitis (*n* = 160) according to extent (E1-E4) and severity (Paris criteria: ever severe vs. never severe). Missing/unknown data are indicated.

### Clinical features

The median time from symptom onset to diagnosis differed among the 3 groups, with UC patients having a median time of 1 year (IQR: 1-2 years), CD patients with a median time of 2 years (IQR: 1-3 years), and IBDU patients with a median time of 1 year (IQR: 1-1 year), and the greatest proportion of patients with disease duration <1 year (Table 3).

**Table 3:** Clinical features, complications, and treatment of IBD by disease type.

Factor	UC	CD	IBDU	*P*-value
N	160	33	84	
**Symptoms start to diagnosis time (years), median (IQR)**	1 (1, 2)	2 (1, 3)	1 (1, 1)	<.001
** = <1 year**	104 (65.0%)	13 (39.4%)	80 (95.2%)	<.001
** 2-5 years**	35 (21.9%)	14 (42.4%)	3 (3.6%)	
** 6-10 years**	6 (3.8%)	5 (15.2%)	0 (0.0%)	
** >10 years**	15 (9.4%)	1 (3.0%)	1 (1.2%)	
**Diagnostic methods**				
** Pathology**	68 (42.5%)	16 (48.5%)	35 (41.7%)	0.79
** Clinical**	90 (56.2%)	17 (51.5%)	23 (27.4%)	<.001
** Endoscopically**	137 (85.6%)	16 (48.5%)	83 (98.8%)	<.001
**CT**	3 (1.9%)	10 (30.3%)	0 (0.0%)	<.001
** Ultrasound**	2 (1.2%)	0 (0.0%)	0 (0.0%)	.48
**Other**	0 (0.0%)	1 (3.0%)	0 (0.0%)	.024
**Clinical characteristics**				
** Abdominal pain**	98 (61.3%)	23 (69.7%)	54 (64.3%)	.64
** Diarrhea**	91 (56.9%)	21 (63.6%)	32 (38.1%)	.007
** Fatigue**	42 (26.2%)	9 (27.3%)	8 (9.5%)	.007
** Constipation**	33 (20.6%)	6 (18.2%)	11 (13.1%)	.35
** Weight loss**	30 (18.8%)	12 (36.4%)	7 (8.3%)	.001
** Anorexia**	21 (13.1%)	4 (12.1%)	2 (2.4%)	.024
** GI bleeding**	62 (38.8%)	12 (36.4%)	13 (15.5%)	<.001
** Vomiting**	13 (8.1%)	9 (27.3%)	2 (2.4%)	<.001
** Not reported**	14 (8.8%)	0 (0.0%)	1 (1.2%)	.016
**Extraintestinal manifestations**				
** Hepatic and pancreatic**	7 (4.4%)	3 (9.1%)	3 (3.6%)	<.001
** Vascular**	1 (0.6%)	6 (18.2%)	0 (0.0%)	<.001
** Articular**	6 (3.8%)	1 (3.0%)	0 (0.0%)	.20
** Dermatologic**	9 (5.6%)	2 (6.1%)	3 (3.6%)	.75
** Ophthalmic**	1 (0.6%)	1 (3.0%)	0 (0.0%)	.21
** Urologic**	1 (0.6%)	2 (6.1%)	0 (0.0%)	.012
** Respiratory**	5 (3.1%)	1 (3.0%)	2 (2.4%)	.95
** None**	128 (80.0%)	13 (39.4%)	78 (92.9%)	<.001
** Unknown/Not reported**	7 (4.4%)	2 (6.1%)	0 (0.0%)	.12
**Non-IBD-related endoscopic findings**				
** Polyps**	31 (19.4%)	5 (15.2%)	16 (19.0%)	.850
** Diverticula**	11 (6.9%)	4 (12.1%)	7 (8.3%)	.590
** None**	62 (38.8%)	13 (39.4%)	51 (60.7%)	.004
** Unknown/Not reported**	55 (34.4%)	9 (27.3%)	12 (14.3%)	.004
**Complications**				
** Cancer of colon**	3 (1.9%)	0 (0.0%)	0 (0.0%)	.330
** Stenosis of colon**	4 (2.5%)	3 (9.1%)	0 (0.0%)	.019
** Enteric fistulas**	0 (0.0%)	8 (24.2%)	0 (0.0%)	<.001
** Unknown/Not reported**	45 (28.1%)	8 (24.2%)	2 (2.4%)	<.001
**Pharmacologic treatment**				
** 5-aminosalicylate**	97 (60.6%)	14 (42.4%)	5 (6.0%)	<.001
** Systemic steroids**	76 (47.5%)	12 (36.4%)	20 (23.8%)	<.001
** Immunomodulators**	24 (15.0%)	13 (39.4%)	0 (0.0%)	<.001
** Calcineurin inhibitors**	3 (1.9%)	0 (0.0%)	0 (0.0%)	.330
**TNF antagonist**	6 (3.8%)	8 (24.2%)	0 (0.0%)	<.001
** No treatment was prescribed**	24 (15.0%)	2 (6.1%)	61 (72.6%)	<.001
**Surgical treatment**				
** Proctocolectomy**	1 (0.6%)	0 (0.0%)	0 (0.0%)	.693
**Colectomy**	3 (1.9%)	3 (9.1%)	1 (1.2%)	.036
**Ileostomy**	2 (1.2%)	2 (6.1%)	0 (0.0%)	.045
**Colostomy**	1 (0.6%)	8 (24.2%)	0 (0.0%)	<.001
**Other**	1 (0.6%)	0 (0.0%)	1 (1.2%)	.772
**Non-surgical treatment**	153 (95.6%)	20 (60.6%)	82 (97.6%)	<.001
**Vital status (at time of data collection)**				<.001
** Alive**	156 (97.5%)	27 (81.8%)	84 (100%)	
** Dead**	4 (2.5%)	6 (18.2%)	0 (0.0%)	

Comparison of disease course and management among UC (*n* = 160), CD (*n* = 33), and IBDU (*n* = 84). Data include diagnostic delay, diagnostic methods, gastrointestinal and extraintestinal manifestations, complications, endoscopic findings, pharmacologic and surgical treatments, and vital status at data collection. Multiple responses were permitted within designated domains (diagnostic methods, clinical characteristics, extraintestinal manifestations, complications and treatment); percentages use the full disease-group denominator unless otherwise noted.

Diagnostic modality utilization varied across IBD subtypes and by country and healthcare setting. Endoscopy was the most frequently used modality, whereas histopathologic confirmation and cross-sectional imaging were less consistently available across centers (Table S2). Endoscopic evaluation was performed in most patients, including 85.6% of UC patients, 48.5% of CD patients, and 98.8% of IBDU patients. Histopathologic confirmation was achieved in 42.5% of UC patients, 48.5% of CD patients, and 41.7% of IBDU patients. Notably, nearly all IBDU patients underwent endoscopy, yet fewer than half had histologic confirmation, raising concerns about the consistency and completeness of diagnostic practices in this group. Table S6 highlights the subgroup with more truly “indeterminate” colitis by restricting to those patients with histopathologic results available.

Among CD patients, only 48.5% underwent endoscopy, and 30.3% had CT imaging, while none received ultrasound. Approximately 42% of CD cases lacked documentation of any objective diagnostic procedure (histopathology or CT), and 12% didn’t have histopathology, CT, or endoscopic data, suggesting potential reliance on clinical judgment alone or incomplete data capture. Use of CT and ultrasound was limited in UC (1.9% and 1.2%, respectively) and absent in IBDU.

Abdominal pain was the most frequently reported symptom, present in 61.3% of UC patients, 69.7% of CD patients, and 64.3% of IBDU patients. Diarrhea was common in both UC (56.9% patients) and CD (63.6% patients) but was reported in only 38.1% of IBDU patients. Constipation occurred in 20.6% of UC patients, 18.2% of CD patients, and 13.1% of IBDU patients. Fatigue was observed in 26.2% of UC patients, 27.3% of CD patients and was less common in IBDU patients at 9.5%. Weight loss was reported by 18.8% of UC patients, 36.4% of CD patients, and 8.3% of IBDU patients. Anorexia was present in 13.1% of UC patients, 12.1% of CD patients, and 2.4% of IBDU patients. Gastrointestinal (GI) bleeding was reported in 38.8% of UC patients, 36.4% of CD patients, and 15.5% of IBDU patients.

Extraintestinal manifestations (EIMs) were observed in a minority of patients. Hepatic and pancreatic manifestations were present in 4.4% of UC patients, 9.1% of CD patients, and 3.6% of IBDU patients. Vascular manifestations (mainly thromboembolism) occurred primarily in CD (18.2%), with only 1 UC patient experiencing vascular manifestation. Articular, dermatologic, ophthalmic, urological, and respiratory manifestations were rare.

Complications in CD patients included enteric fistulas (24.2%) and intestinal strictures, with a concentration in Honduras, where 33.3% of CD patients had enteric fistulas compared to 11.1% in El Salvador and 16.7% in Guatemala. Additional complications observed in the cohort included GI bleeding and colonic stenosis. Colon cancer was identified in 3 UC patients (1.9%), but no CD or IBDU patients. Colon stenosis was more common in CD, seen in 9.1% patients, compared to 2.5% in UC patients and none in IBDU (Table 3).

### Treatment

The most common treatment for UC was 5-aminosalicylic acid (5-ASA), used by 60.6% of patients, followed by systemic steroids (47.5%) and immunomodulators (15.0%). In CD, 5-ASA use was also prevalent at 42.4%, followed by immunomodulators (39.4%), systemic steroids (36.4%), and tumor necrosis factor (TNF) antagonists (24.2%). For patients with IBDU, a high proportion (72.6%) received no treatment, while only 23.8% were treated with systemic steroids and 6.0% with 5-ASA. Surgical treatment was uncommon among patients with UC (0.6%) and IBDU but was substantially more frequent among patients with CD with 39.4% undergoing at least one IBD-related surgical procedure. Colectomy was performed in 3 patients with CD (9.1%), 3 patients with UC (1.9%), and 1 patient with IBDU (1.2%). Diverting colostomy was performed in 8 patients with CD (24.2%) and 1 patient with UC (0.6%). Ileostomy was performed in 2 patients with CD (6.1%) and 2 patients with UC (1.2%). No ostomies were reported among patients with IBDU. At the time of data collection, 6 patients with CD, 4 with UC, and no patients with IBDU were reported deceased.

Treatment was influenced by both country and care setting. Guatemalan patients received 5-ASA agents only 5.9% of the time compared to 51%-54% in Honduras and El Salvador, but in turn used steroids much more frequently (72.1% vs 42.9% in Honduras and 20.9% in El Salvador, *P*<.001, Supplement 2). Private clinic patients were also less likely to receive steroids (12.8% vs 56.0%, *P*<.001), immunomodulators (1.8% vs 20.8%, *P*<.001), or anti-TNFs (0% vs 8.3%, *P*=.002), and more likely to receive no treatment (50.5% vs 19.0%, *P*<.001). Surgical interventions were also less common in private clinics than in hospital-based practices (4.5% vs 10.7%, *P*=.017), and more frequently performed in Honduras (20%) than Guatemala (4.4%) or El Salvador (4.3%, *P*<.001).

## Discussion

To the best of our knowledge, this study presents the largest IBD cohort in Northern Central America, a region previously excluded from systematic assessment of IBD in Latin America. The most recent data in Latin America comes from a pair of systematic reviews,^5^^,^^15^ with included study periods from 1980 to 2015, but all non-Brazilian studies from earlier than 2009. In these reviews, the incidence rates of UC ranged from 0.0 to 8.00 per 100,000 person-years, with a prevalence of 0.99 to 82.2 per 100,000 inhabitants. For CD, the incidence rates ranged from 0.24 to 6.83 per 100,000 person-years, and the prevalence ranged from 0.24 to 24.1 per 100,000 inhabitants.^5^^,^^15^ However, no data included Northern Central America, the core of Latin America LMICs.^14^ Many of these population-level estimates, such as those derived from the Global Burden of Disease framework, also rely on modeled data from mortality registries, administrative datasets, and statistical approaches rather than direct patient-level clinical verification. Our study addresses this gap by providing data based on manually reviewed clinical records collected from healthcare institutions in three Central American countries. Consequently, while this cohort likely represents only a portion of the true IBD population in Northern Central America, it provides important insight into clinical presentation, diagnostic patterns, and treatment access in real-world healthcare settings.

Our findings revealed a higher prevalence of UC compared to CD, which aligns with similar studies from other developing or newly industrialized countries. In Colombia, the incidence rate of UC increased from 5.59 to 6.30 per 100,000 persons between 2010 and 2017, while CD rates remained stable.^16^ Similarly, UC is more prevalent than CD in countries like Venezuela, Mexico, Cuba, Ecuador, and Uruguay, while CD is more common in Puerto Rico, the Dominican Republic, and Peru.^17^ These patterns suggest regional variations in disease distribution across Latin America, which may be influenced by factors such as genetic predisposition and environmental exposures. Incidence is increasing across all regions studied, and the concurrent increasing Westernization may eventually lead to a decreasing UC: CD ratio as has been seen in fully industrialized nations.

In our cohort, the median age of IBD diagnosis ranged from 42.3 to 56.9 years, later than the U.S. median age of 32.7 years.^18^ While the older age at diagnosis might suggest delayed healthcare access in some settings, our findings of a short period from symptom onset to diagnosis indicate that, once patients engaged with the healthcare system, they accessed diagnostic care relatively quickly despite potential socioeconomic challenges. However, our data on methods of diagnosis suggest persistent significant resource limitations for our cohort, such as the findings that less than half of Crohn’s patients underwent endoscopic evaluation, the minority of all IBD patients had histopathology examinations, and even fewer had radiologic examinations.

In other settings, approximately 5% of patients with IBD are diagnosed with IBDU because of inability to differentiate between CD and UC.^19^ Our study found a much higher percentage (30.3%) categorized as having IBDU. A substantial proportion of these patients were not receiving treatment in our data set, and there was striking variation in IBDU prevalence by country and practice setting, with a much higher proportion of IBDU cases in El Salvador (40.3%) and Guatemala (38.2%) compared to Honduras (2.9%), as well as private clinics (51.4%) compared to hospital-based settings (16.7%). This suggests that the increased use of the IBDU diagnosis may be related to regional or other limitations in access to specialized diagnostics, and/or physician comfort in making a definitive IBD diagnosis. The substantial proportion of overall IBDU patients not receiving treatment suggests that some of these may not have been true IBD at all. Infectious, ischemic, and medication-induced colitis may present similarly to IBDU, particularly in LMICs where comprehensive diagnostic tools are less accessible. However, in the primary cohort, 27.1% of patients with IBDU received no treatment, and in the sensitivity analysis, no patients with histopathologically confirmed IBDU received immunomodulators or TNF antagonist therapy, suggesting that diagnostic uncertainty may contribute to delayed or inadequate management. These findings highlight the need for standardized diagnostic criteria, improved access to histopathology, and specialized training for clinicians in Latin America to enhance diagnostic accuracy and ensure timely treatment initiation.

EIMs were observed in 17.7% of all IBD patients in our study, with the highest frequency in CD (54.5%), followed by UC (15.6%) and IBDU (7.1%). Musculoskeletal issues (primarily arthropathy) are the most frequently observed EIM in patients with IBD globally, occurring in 20%-30% of patients during follow-up of European cohorts.^20–22^ Our study found a lower prevalence of articular EIMs, under 3% of patients, and more frequently in UC (3.8%) and CD (3.0%) than IBDU (0%). The prevalence of mucocutaneous manifestations, most commonly erythema nodosum, pyoderma gangrenosum, and aphthous stomatitis, was approximately 6%, also on the lower end of ranges reported in Western populations (5%-50%).^23^

Our study also highlights the limited access to certain treatments in LMICs, with notably high steroid use in Central America compared to high-income countries and even other Latin American regions.^17^ The discrepancy found between the lower treatment rates in private clinic patients may be due to differences in disease severity, provider experience, access to care, or insurance barriers, as well as limited infrastructure and follow-up. Biologics like anti-TNF therapies, while increasing in use, remain challenging to access, which likely impacts hospitalization rates and IBD complications. Although mortality appeared higher among patients with CD, cause-of-death data were not available at the time of analysis, limiting interpretation of this finding.

The limited use of advanced therapies observed in this cohort likely reflects structural healthcare barriers rather than differences in disease severity. Financial barriers, limited medication availability, fragmented healthcare access, and loss to follow-up likely contributed to the treatment patterns observed in this cohort. There is a need for new strategies to improve access to advanced therapies in low-resource settings of Central America.

### Limitations

The limitations of this study include its retrospective design, which limits the completeness of the data set. This and reliance on diagnostic codes introduce potential imprecision in diagnoses, including the higher-than-expected prevalence of IBDU. The limited data on IBD in Latin American countries and challenges in accessing advanced diagnostic methods in LMICs may contribute to both the high number of IBDU diagnoses and the later age of diagnosis observed in this cohort. This raises concerns about the accuracy of IBDU classification in these settings, including the possibility that some cases may not represent true IBD. Access to diagnostic testing was heterogeneous across participating centers. Histopathologic confirmation, cross-sectional imaging, and repeat endoscopic evaluation were not uniformly available, which may have contributed to diagnostic uncertainty not only for patients classified as IBD-unclassified but also for some patients diagnosed with UC or CD. Table S5 includes a table of main characteristics and outcomes, only including UC and CD. The selection of only a few clinical sites, mostly urban academic centers with only two private practices (also urban), limits generalizability and may introduce selection bias. These patients are more likely to have severe phenotypes and to have had the appropriate diagnostics and been offered therapy than a nationally representative sample. The 10-year period of this study also overlapped with significant advances in IBD therapeutics, such that practice patterns may have varied significantly over the course of the study.

Despite these limitations, this study provides invaluable insight into the epidemiology and clinical characteristics of IBD in Central America, including delays in diagnosis and treatment even in referral centers, thereby informing the development of more effective diagnostic strategies for patients with IBD in this region.

## Conclusion

Our study contributes essential data on the clinical characteristics and risk factors for IBD in Central America. Our findings underscore a higher prevalence of UC than CD, a later median age of diagnosis, and challenges in accessing precise diagnostics as well as advanced treatment. This epidemiological snapshot can inform future public health interventions and improve IBD management in Northern Central America, ultimately aiming to enhance the quality of care and health outcomes for this population.

## Supplementary Material

otag106_Supplementary_Data

## Data Availability

The data underlying this article are not publicly available due to patient privacy and confidentiality considerations. De-identified data may be made available by the corresponding author upon reasonable request and subject to applicable institutional approvals and data-sharing agreements.

## Data access statement

Data will be available as requested.
