# Supplementary material for: Clinical characteristics and phenotypes of inflammatory bowel disease in Northern Central America: initial results from the Northern Central America Inflammatory Bowel Disease Registry
Source: Crohns Colitis 360. 2026 Oct 21;8(4):otag106. doi: 10.1093/crocol/otag106 (PMC13716489; doi:10.1093/crocol/otag106)
Supplement: Supplementary file 1 [file otag106_supplementary_data.docx]

Supplement Table 1. Medical record search descriptors

| Search Strategy | Description |
| --- | --- |
| ICD-10 codes | Records were screened using the tenth revision of the international classification of diseases (ICD-10) codes for inflammatory bowel disease, selecting codes K50 Group (for Crohn’s Disease), K51 Group (for UC), and K52 Group (for IBDU). |
| Diagnosis nomenclature | Inflammatory Bowel Disease, IBD, Crohn’s Disease, Ulcerative Colitis, and inflammatory bowel disease unclassified. |
| Diagnosis Nomenclature Spanish | Enfermedad Inflamatoria Intestinal, EII, Enfermedad de Crohn, Colitis Ulcerativa, Colitis Ulcerosa, Enfermedad Inflamatoria Intestinal no clasificada and Colitis Indeterminada. |

Search strategy used to identify cases of inflammatory bowel disease (IBD) across participating sites. Records were screened using ICD-10 codes for Crohn’s disease (K50 group), ulcerative colitis (K51 group), and IBD unclassified (K52 group). Diagnosis nomenclature included both English and Spanish terms commonly used in clinical practice.

**Supplement Table 2. Characteristics of IBD patients by country.**

|  | El Salvador | Guatemala | Honduras | p-value |
| --- | --- | --- | --- | --- |
|  | 139 | 68 | 70 |  |
| **Diagnosis** |  |  |  | <0.001 |
| Ulcerative Colitis | 74 (53.2%) | 36 (52.9%) | 50 (71.4%) |  |
| Crohn’s Disease | 9 (6.5%) | 6 (8.8%) | 18 (25.7%) |  |
| IBDU | 56 (40.3%) | 26 (38.2%) | 2 (2.9%) |  |
| **Age at Diagnosis, median (IQR)** | 52.9 (38.9, 64.6) | 46.9 (26.8, 58.4) | 35.7 (20.2, 45.9) | <0.001 |
| <18 | 0 (0.0%) | 6 (8.8%) | 12 (17.1%) | <0.001 |
| 18-24 | 5 (3.6%) | 10 (14.7%) | 5 (7.1%) |  |
| 25-44 | 43 (30.9%) | 16 (23.5%) | 20 (28.6%) |  |
| 45-64 | 56 (40.3%) | 27 (39.7%) | 15 (21.4%) |  |
| >65 | 33 (23.8%) | 9 (13.3%) | 2 (2.9%) |  |
| Unknown or not reported | 2 (1.4%) | 0 (0.0%) | 16 (22.9%) |  |
| **Sex** |  |  |  | 0.088 |
| Female | 81 (58.2%) | 41 (60.3%) | 31 (44.3%) |  |
| Male | 55 (39.6%) | 26 (38.2%) | 38 (54.3%) |  |
| Unknown or not reported | 3 (2.2%) | 1 (1.5%) | 1 (1.4%) |  |
| **Race** |  |  |  | <0.001 |
| White | 1 (0.7%) | 12 (17.6%) | 22 (31.4%) |  |
| Black | 0 (0.0%) | 1 (1.5%) | 1 (1.4%) |  |
| More than one race | 138 (99.3%) | 7 (10.3%) | 34 (48.6%) |  |
| Unknown or not reported | 0 (0.0%) | 48 (70.6%) | 13 (18.6%) |  |
| **Marital Status** |  |  |  | <0.001 |
| Married or living together | 39 (28.1%) | 35 (51.5%) | 24 (34.3%) |  |
| Single | 67 (48.2%) | 0 (0.0%) | 34 (48.6%) |  |
| Widow | 28 (20.1%) | 33 (48.5%) | 12 (17.1%) |  |
| Other | 5 (3.6%) | 0 (0.0%) | 0 (0.0%) |  |
| **Geographic Setting** |  |  |  | 0.006 |
| Rural | 40 (28.8%) | 34 (50.0%) | 20 (28.6%) |  |
| Urban | 99 (71.2%) | 34 (50.0%) | 50 (71.4%) |  |
| **Education** |  |  |  | <0.001 |
| Middle School Incomplete | 2 (1.4%) | 14 (20.6%) | 5 (7.1%) |  |
| Middle School Complete | 1 (0.7%) | 28 (41.2%) | 9 (12.9%) |  |
| Highschool Incomplete | 10 (7.2%) | 9 (13.2%) | 4 (5.7%) |  |
| Highschool Complete | 15 (10.8%) | 11 (16.2%) | 18 (25.7%) |  |
| University no-degree | 11 (7.9%) | 1 (1.5%) | 2 (2.9%) |  |
| University Degree | 7 (5.0%) | 5 (7.4%) | 5 (7.1%) |  |
| Unknown or not reported | 93 (66.9%) | 0 (0.0%) | 27 (38.6%) |  |
| **Sedentarism** | 118 (84.9%) | 26 (38.2%) | 24 (34.3%) | <0.001 |
| Unknown or not reported | 21 (15.1%) | 42 (61.8%) | 46 (65.7%) |  |
| **Alcohol consumption** | 12 (8.6%) | 24 (35.3%) | 12 (17.1%) | <0.001 |
| Unknown or not reported | 127 (91.4%) | 44 (64.7%) | 58 (82.9%) |  |
| **Comorbidities** |  |  |  |  |
| Cardiovascular | 37 (26.6%) | 18 (26.5%) | 6 (8.6%) | 0.007 |
| Metabolic | 19 (13.7%) | 12 (17.6%) | 8 (11.4%) | 0.57 |
| Infectious | 0 (0.0%) | 6 (8.8%) | 2 (2.9%) | 0.002 |
| Chronic/non-infectious | 18 (12.9%) | 7 (10.3%) | 15 (21.4%) | 0.14 |
| Neurological | 0 (0.0%) | 0 (0.0%) | 2 (2.9%) | 0.051 |
| Cancer | 0 (0.0%) | 5 (7.4%) | 1 (1.4%) | 0.003 |
| Other Autoimmune | 3 (2.2%) | 1 (1.5%) | 2 (2.9%) | 0.86 |
| None | 67 (48.2%) | 32 (47.1%) | 32 (45.7%) | 0.94 |
| **Tuberculosis** | 3 (2.2%) | 2 (3.0%) | 1 (1.5%) | 0.83 |
| **Symptoms start to Diagnosis time (years), median (IQR)** | 1 (1, 1) | 1 (1, 1) | 3 (1, 10) | <0.001 |
| =<1 year | 116 (83.5%) | 56 (82.4%) | 25 (35.7%) | <0.001 |
| 2-5 years | 23 (16.5%) | 12 (17.6%) | 17 (24.3%) |  |
| 6-10 years | 0 (0.0%) | 0 (0.0%) | 11 (15.7%) |  |
| >10 years | 0 (0.0%) | 0 (0.0%) | 17 (24.3%) |  |
| **Clinical Characteristics** |  |  |  |  |
| Abdominal Pain | 87 (62.6%) | 46 (67.6%) | 42 (60.0%) | 0.63 |
| Diarrhea | 47 (33.8%) | 56 (82.4%) | 41 (58.6%) | <0.001 |
| Fatigue | 20 (14.4%) | 24 (35.3%) | 15 (21.4%) | 0.003 |
| Constipation | 39 (28.1%) | 4 (5.9%) | 7 (10.0%) | <0.001 |
| Weight Loss | 14 (10.1%) | 11 (16.2%) | 24 (34.3%) | <0.001 |
| Anorexia | 8 (5.8%) | 13 (19.1%) | 6 (8.6%) | 0.009 |
| GI Bleeding | 38 (27.3%) | 18 (26.5%) | 31 (44.3%) | 0.027 |
| Vomiting | 2 (1.4%) | 2 (2.9%) | 20 (28.6%) | <0.001 |
| Not Reported | 0 (0.0%) | 0 (0.0%) | 15 (21.4%) | <0.001 |
| **Extraintestinal Manifestations** |  |  |  |  |
| Hepatic and pancreatic | 4 (2.9%) | 5 (7.4%) | 4 (5.7%) | 0.32 |
| Renal | 0 (0.0%) | 4 (5.9%) | 11 (15.7%) | <0.001 |
| Vascular | 0 (0.0%) | 0 (0.0%) | 7 (10.0%) | <0.001 |
| Articular | 6 (4.3%) | 0 (0.0%) | 1 (1.4%) | 0.14 |
| Dermatologic | 7 (5.0%) | 2 (2.9%) | 5 (7.1%) | 0.53 |
| Ophthalmic | 1 (0.7%) | 0 (0.0%) | 1 (1.4%) | 0.61 |
| Urologic | 0 (0.0%) | 0 (0.0%) | 3 (4.3%) | 0.011 |
| Respiratory | 0 (0.0%) | 4 (5.9%) | 4 (5.7%) | 0.016 |
| None | 119 (85.6%) | 60 (88.2%) | 40 (57.1%) | <0.001 |
| Unknown/Not Reported | 2 (1.4%) | 1 (1.5%) | 6 (8.6%) | 0.015 |
| **Complications** |  |  |  |  |
|  |  |  |  |  |
| Colon Cancer | 2 (1.4%) | 0 (0.0%) | 1 (1.4%) | 0.61 |
| Colon stenosis | 2 (1.4%) | 3 (4.4%) | 2 (2.9%) | 0.43 |
| Cholangitis | 0 (0.0%) | 1 (1.5%) | 0 (0.0%) | 0.21 |
| Enteric fistulas | 1 (0.7%) | 1 (1.5%) | 6 (8.6%) | 0.007 |
| None | 98 (70.5%) | 53 (77.9%) | 27 (38.6%) | <0.001 |
| Unknown/Not Reported | 35 (25.2%) | 2 (2.9%) | 18 (25.7%) | <0.001 |
| **Pharmacologic Treatment** |  |  |  |  |
| 5-ASA or sulfasalazine | 76 (54.7%) | 4 (5.9%) | 36 (51.4%) | <0.001 |
| Systemic Steroids | 29 (20.9%) | 49 (72.1%) | 30 (42.9%) | <0.001 |
| Immunomodulators | 8 (5.8%) | 15 (22.1%) | 14 (20.0%) | <0.001 |
| Calcineurin Inhibitors | 0 (0.0%) | 1 (1.5%) | 2 (2.9%) | 0.16 |
| TNF antagonist | 4 (2.9%) | 7 (10.3%) | 3 (4.3%) | 0.069 |
| No treatment was prescribed | 56 (40.3%) | 12 (17.6%) | 19 (27.1%) | 0.003 |
| **Surgical Treatment** |  |  |  |  |
| Proctocolectomy | 1 (0.7%) | 0 (0.0%) | 0 (0.0%) | 0.61 |
| Colectomy | 3 (2.1%) | 1 (1.5%) | 3 (4.3%) | 0.53 |
| Ileostomy | 1 (0.7%) | 2 (2.9%) | 1 (1.4%) | 0.45 |
| Colostomy Bag | 0 (0.0%) | 0 (0.0%) | 9 (12.9%) | <0.001 |
| Other | 1 (0.7%) | 0 (0.0%) | 1 (1.4%) | 0.61 |
| Non-surgical treatment | 133 (95.7%) | 65 (95.6%) | 56 (80.0%) | <0.001 |
| Diagnosis Modality | |  |  |  |
| Pathology | 66 (47.5%) | 9 (13.2%) | 44 (62.9%) | <0.001 |
| Clinical | 39 (28.1%) | 49 (72.1%) | 42 (60.0%) | <0.001 |
| Endoscopy | 124 (89.2%) | 67 (98.5%) | 45 (64.3%) | <0.001 |
| CT | 3 (2.2%) | 0 (0.0%) | 10 (14.3%) | <0.001 |
| Ultrasound | 0 (0.0%) | 0 (0.0%) | 2 (2.9%) | 0.051 |
| Other | 1 (0.7%) | 0 (0.0%) | 0 (0.0%) | 0.61 |

Baseline sociodemographic and clinical characteristics of patients with IBD from El Salvador (n=139), Guatemala (n=68), and Honduras (n=70). Data include diagnosis distribution, age at diagnosis, sex, race, marital status, geographic setting, education, lifestyle factors, comorbidities, diagnostic delay, clinical features, extraintestinal manifestations, complications, and medical/surgical treatment. Comparisons between countries are shown with corresponding p-values.

**Supplement Table 3: Characteristics of IBD Patients by Type of Practice**

| **Characteristic** | **University Hospital Practice (N=168)** | **Private Practice (N=109)** | **p-value** |
| --- | --- | --- | --- |
| **Diagnosis** |  |  | <0.001 |
| Ulcerative Colitis | 111 (66.1%) | 49 (44.9%) |  |
| Crohn’s Disease | 29 (17.3%) | 4 (3.7%) |  |
| IBDU | 28 (16.7%) | 56 (51.4%) |  |
| **Geographic Setting** |  |  | 0.43 |
| Rural | 54 (32.1%) | 40 (36.7%) |  |
| Urban | 114 (67.9%) | 69 (63.3%) |  |
| **Time for Diagnosis (Median, IQR)** | 1 (1,2) | 1 (1,1) | <0.001 |
| **Pharmacologic Treatment** |  |  |  |
| 5-aminosalicylate | 64 (38.1%) | 52 (47.7%) | 0.11 |
| Systemic Steroids | 94 (56.0%) | 14 (12.8%) | <0.001 |
| Immunomodulators | 35 (20.8%) | 2 (1.8%) | <0.001 |
| Calcineurin Inhibitors | 3 (1.8%) | 0 (0.0%) | 0.16 |
| TNF antagonist | 14 (8.3%) | 0 (0.0%) | 0.002 |
| No treatment was prescribed | 32 (19.0%) | 55 (50.5%) | <0.001 |
| **Surgical Treatment** |  |  |  |
| Proctocolectomy | 0 (0.0%) | 1 (0.9%) | 0.21 |
| Colectomy | 5 (3.0%) | 2 (1.8%) | 0.55 |
| Ileostomy | 3 (1.8%) | 1 (0.9%) | 0.55 |
| Colostomy | 9 (5.4%) | 0 (0.0%) | 0.014 |
| Other | 1 (0.6%) | 1 (0.9%) | 0.76 |
| Non-surgical treatment | 150 (89.3%) | 104 (95.5%) | 0.017 |
| **Vital Status** |  |  | 0.009 |
| Alive | 158 (94.0%) | 109 (100.0%) |  |
| Death | 10 (6.0%) | 0 (0.0%) |  |
| **Digestive Tract Disease / Endoscopic findings** |  |  |  |
| Polyps | 21 (12.5%) | 31 (28.4%) | <0.001 |
| Diverticula | 9 (5.4%) | 13 (11.9%) | 0.048 |
| None | 78 (46.4%) | 48 (44.0%) | 0.700 |
| Unknown/Not reported | 57 (33.9%) | 19 (17.4%) | 0.003 |

*Comparison of patients with IBD seen at university hospitals (n=168) versus private practices (n=109). Variables include diagnosis, geographic setting, time to diagnosis, pharmacologic and surgical treatments, vital status, and endoscopic findings. p-values indicate statistical comparisons between practice types.*

Supplement Figure 1.

**Supplementary Table 4**. **Key patient characteristics by diagnosis period**

| **Characteristic** | **2014–2019 (n=43)** | **2015–2019 (n=125)** | **2020–2024 (n=96)** | **p-value** |
| --- | --- | --- | --- | --- |
| **Diagnosis type, n (%)** |  |  |  | 0.38 |
| UC | 29 (67.4) | 71 (56.8) | 55 (57.3) |  |
| CD | 4 (9.3) | 13 (10.4) | 16 (16.7) |  |
| IBDU | 10 (23.3) | 41 (32.8) | 25 (26.0) |  |
| **Country, n (%)** |  |  |  | <0.001 |
| El Salvador | 8 (18.6) | 59 (47.2) | 66 (68.8) |  |
| Guatemala | 18 (41.9) | 29 (23.2) | 15 (15.6) |  |
| Honduras | 17 (39.5) | 37 (29.6) | 15 (15.6) |  |
| **Age, median (IQR), years** | 47.1 (36.4–59.1) | 44.3 (30.0–62.5) | 48.3 (35.0–59.5) | 0.74 |
| **Sex, n (%)** |  |  |  | 0.83 |
| Female | 25 (58.1) | 65 (52.0) | 50 (52.1) |  |
| Male | 18 (41.9) | 58 (46.4) | 43 (44.8) |  |
| **Geographic setting, n (%)** |  |  |  | 0.003 |
| Urban | 23 (53.5) | 77 (61.6) | 76 (79.2) |  |
| Rural | 20 (46.5) | 48 (38.4) | 20 (20.8) |  |
| **Alcohol consumption (yes), n (%)** | 16 (37.2) | 18 (14.4) | 13 (13.5) | 0.001 |
| **Chronic/non-infectious comorbidities (yes), n (%)** | 2 (4.7) | 17 (13.6) | 20 (20.8) | 0.040 |
| **Time from symptoms to diagnosis, median (IQR), years** | 1 (1–10) | 1 (1–2) | 1 (1–1) | 0.017 |
| **Time to diagnosis ≤1 year, n (%)** | 26 (60.5) | 86 (68.8) | 73 (76.0) | <0.001 |
| **5-ASA / sulfasalazine use (yes), n (%)** | 8 (18.6) | 55 (44.0) | 52 (54.2) | <0.001 |

**Supplement table 5. Key patient characteristics sensitivity analysis only including UC and CD.**

| **Characteristic** | **UC (n=160)** | **CD (n=33)** | **p-value** |
| --- | --- | --- | --- |
| **Age, median (IQR), years** | 42.3 (30.0–54.2) | 47.8 (38.7–62.9) | 0.062 |
| **Female sex, n (%)** | 89 (55.6) | 12 (36.4) | 0.052 |
| **Race, n (%)** |  |  | <0.001 |
| White | 15 (9.4) | 19 (57.6) |  |
| Multiracial | 111 (69.4) | 11 (33.3) |  |
| Unknown/not reported | 33 (20.6) | 2 (6.1) |  |
| **Urban residence, n (%)** | 115 (71.9) | 28 (84.8) | 0.12 |
| **No comorbidities, n (%)** | 83 (51.9) | 5 (15.2) | <0.001 |
| **Time from symptoms to diagnosis, median (IQR), years** | 1 (1–2) | 2 (1–3) | 0.021 |
| **Diagnosis within ≤1 year, n (%)** | 104 (65.0) | 13 (39.4) | 0.002 |
| **Weight loss at presentation, n (%)** | 30 (18.8) | 12 (36.4) | 0.026 |
| **Vomiting at presentation, n (%)** | 13 (8.1) | 9 (27.3) | 0.002 |
| **Renal extraintestinal manifestations, n (%)** | 7 (4.4) | 8 (24.2) | <0.001 |
| **Vascular extraintestinal manifestations, n (%)** | 1 (0.6) | 6 (18.2) | <0.001 |
| **Urologic extraintestinal manifestations, n (%)** | 1 (0.6) | 2 (6.1) | 0.022 |
| **No extraintestinal manifestations, n (%)** | 128 (80.0) | 13 (39.4) | <0.001 |
| **Immunomodulator use, n (%)** | 24 (15.0) | 13 (39.4) | 0.001 |
| **TNF antagonist use, n (%)** | 6 (3.8) | 8 (24.2) | <0.001 |
| **Major surgery related to IBD, n (%)** | 1 (0.6) | 8 (24.2) | <0.001 |

Supplement table 6. Key patient characteristics sensitivity analysis only included histologically confirmed IBDU.

| **Characteristic** | **UC (n=160)** | **CD (n=33)** | IBDU* (n=35) | **p-value** |
| --- | --- | --- | --- | --- |
| **Age, median (IQR), years** | 42.3 (30.0–54.2) | 47.8 (38.7–62.9) | 54.1 (37.9-68.8) | 0.005 |
| **Female sex, n (%)** | 89 (55.6) | 12 (36.4) | 23 (67.6) | 0.044 |
| **Race, n (%)** |  |  |  | <0.001 |
| White | 15 (9.4) | 19 (57.6) | 0 (0.0) |  |
| Multiracial | 111 (69.4) | 11 (33.3) | 34 (97.1) |  |
| Unknown/not reported | 33 (20.6) | 2 (6.1) | 1 (2.9) |  |
| **Urban residence, n (%)** | 115 (71.9) | 28 (84.8) | 15 (42.9) | <0.001 |
| **No comorbidities, n (%)** | 83 (51.9) | 5 (15.2) | 20 (57.1) |  |
| **Time from symptoms to diagnosis, median (IQR), years** | 1 (1–2) | 2 (1–3) | 1 (1-1) | <0.001 |
| **Diagnosis within ≤1 year, n (%)** | 104 (65.0) | 13 (39.4) | 34 (97.1) | <0.001 |
| **Weight loss at presentation, n (%)** | 30 (18.8) | 12 (36.4) | 1 (2.9) | 0.002 |
| **Vomiting at presentation, n (%)** | 13 (8.1) | 9 (27.3) | 0 (0.0) | <0.001 |
| **Renal extraintestinal manifestations, n (%)** | 7 (4.4) | 8 (24.2) | 0 (0.0) | <0.001 |
| **Vascular extraintestinal manifestations, n (%)** | 1 (0.6) | 6 (18.2) | 0 (0.0) | <0.001 |
| **Urologic extraintestinal manifestations, n (%)** | 1 (0.6) | 2 (6.1) | 0 (0.0) | <0.034 |
| **No extraintestinal manifestations, n (%)** | 128 (80.0) | 13 (39.4) | 34 (97.1) | <0.001 |
| **Immunomodulator use, n (%)** | 24 (15.0) | 13 (39.4) | 0 (0.0) | <0.001 |
| **TNF antagonist use, n (%)** | 6 (3.8) | 8 (24.2) | 0 (0.0) | <0.001 |
| **Major surgery related to IBD, n (%)** | 1 (0.6) | 8 (24.2) | 0 (0.0) | <0.001 |

*IBDU only with histopathological confirmation.
